# Validity of the Rapid Eating Assessment for Patients for assessing dietary patterns in NCAA athletes

**DOI:** 10.1186/s12970-014-0042-y

**Published:** 2014-08-15

**Authors:** Jonathan M Kurka, Matthew P Buman, Barbara E Ainsworth

**Affiliations:** 1 Exercise and Wellness Program, School of Nutrition and Health Promotion, Arizona State University, 500 N Third Street, Phoenix 85004, AZ, USA

**Keywords:** Athlete, Nutrition, Factor analysis

## Abstract

**Background:**

Athletes may be at risk for developing adverse health outcomes due to poor eating behaviors during college. Due to the complex nature of the diet, it is difficult to include or exclude individual food items and specific food groups from the diet. Eating behaviors may better characterize the complex interactions between individual food items and specific food groups. The purpose was to examine the Rapid Eating Assessment for Patients survey (REAP) as a valid tool for analyzing eating behaviors of NCAA Division-I male and female athletes using pattern identification. Also, to investigate the relationships between derived eating behavior patterns and body mass index (BMI) and waist circumference (WC) while stratifying by sex and aesthetic nature of the sport.

**Methods:**

Two independent samples of male (n = 86; n = 139) and female (n = 64; n = 102) collegiate athletes completed the REAP in June-August 2011 (n = 150) and June-August 2012 (n = 241). Principal component analysis (PCA) determined possible factors using wave-1 athletes. Exploratory (EFA) and confirmatory factor analyses (CFA) determined factors accounting for error and confirmed model fit in wave-2 athletes. Wave-2 athletes’ BMI and WC were recorded during a physical exam and sport participation determined classification in aesthetic and non-aesthetic sport. Mean differences in eating behavior pattern score were explored. Regression models examined interactions between pattern scores, participation in aesthetic or non-aesthetic sport, and BMI and waist circumference controlling for age and race.

**Results:**

A 5-factor PCA solution accounting for 60.3% of sample variance determined fourteen questions for EFA and CFA. A confirmed solution revealed patterns of Desserts, Healthy food, Meats, High-fat food, and Dairy. Pattern score (mean ± SE) differences were found, as non-aesthetic sport males had a higher (better) Dessert score than aesthetic sport males (2.16 ± 0.07 vs. 1.93 ± 0.11). Female aesthetic athletes had a higher score compared to non-aesthetic female athletes for the Dessert (2.11 ± 0.11 vs. 1.88 ± 0.08), Meat (1.95 ± 0.10 vs. 1.72 ± 0.07), High-fat food (1.70 ± 0.08 vs. 1.46 ± 0.06), and Dairy (1.70 ± 0.11 vs. 1.43 ± 0.07) patterns.

**Conclusions:**

REAP is a construct valid tool to assess dietary patterns in college athletes. In light of varying dietary patterns, college athletes should be evaluated for healthful and unhealthful eating behaviors.

## Background

Young adults with unhealthful eating behaviors are at risk for poor health outcomes [1]. Those involved in team sports requiring strength and power (i.e., football) may be at risk for being overweight and for developing chronic conditions [2]. Approximately 50% of amateur football linemen may be obese (body mass index ≥ 30) [2] and more likely to have insulin resistance compared to their non-obese counterparts [3]. Healthful eating behaviors should be encouraged in young adulthood [4]. The college lifestyle includes barriers to healthful eating behaviors such as limited cooking skills and limited finances leading to meal skipping or frequent snacking on readily accessible unhealthful food [5],[6]. College athletes are particularly vulnerable to poor eating habits due to the added demands of competitive sport and the need for nutritional services and education on healthful dietary habits in members of athletic teams and sports is evident [6].

Disease risk factors associated with diet are often attributed to increased intake or lack of consumption of singular nutrients (e.g., saturated fat, dietary fiber) or food groups (e.g., fruits and vegetables) [7]. However, including or excluding individual food items or food groups to or from the diet is difficult due to its complex nature. Because of these complex interactions, dietary habits are becoming increasingly characterized as latent variables or constructs. Latent variable analysis is the emerging standard of measuring dietary habits or “dietary patterns” using pattern identification protocols (i.e. cluster and factor analysis) [8]. Latent variable analysis has contributed to the understanding of dietary composition related to health outcomes [9], as healthful dietary patterns reduce risks for CVD markers [10].

Our purpose was to determine construct validity of the nutrition component of the Rapid Eating and Activity Assessment for Patients (REAP) to describe dietary patterns of NCAA Division-I athletes using pattern identification protocol. Secondly, dietary pattern scores were examined in males and females between sport types, with the hypothesis that athletes in sports where success is partially dependent on an amenable physique (e.g., gymnastics) exhibit different scores than athletes in sports where an appealing physique has no impact on success (e.g., baseball/softball). Lastly, we explored whether dietary pattern score was a predictor of CVD markers of body mass index (BMI) and waist circumference.

## Methods

Data were obtained during two separate waves of collection, June-August 2011 (n = 150) and June-August 2012 (n = 241). In each wave, convenience samples of male and female NCAA Division-I athletes were asked to complete an informed consent and the REAP either immediately before or after a pre-participation physical examination. The protocol was approved by the University Office of Research Integrity and Assurance. Demographic information was approved for extraction from the athlete’s electronic medical record (EMR) by the lead researcher and included sex, age, race/ethnicity, and sport.

Data from the first wave (n = 150) of completed REAP surveys identified possible dietary patterns using principal components analysis (PCA). Data from the second wave (n = 241) confirmed dietary patterns using exploratory (EFA) and confirmatory (CFA) factor analysis. Mean differences in dietary pattern scores of athletes after stratifying by gender and the aesthetic nature of the sport were compared. The interactive role of dietary pattern score x aesthetic nature of the sport on markers of CVD (BMI and waist circumference) was examined within these subpopulations.

### Measurements

The REAP was originally developed to evaluate the dietary behaviors with the goal to identify a comprehensive nutritional profile [11]. The original survey includes 27 questions assessing the eating frequency of breakfast and meals not prepared in the home, intake of whole grains, fruits and vegetables, calcium-rich foods, saturated fat and cholesterol, sugar-rich food and beverages, sodium, alcohol beverages, and physical activity level. The survey takes approximately 10 minutes to complete and is written at the sixth-grade reading level. Practicing physicians consider the survey a feasible tool to assess patients’ dietary habits and it is valid against the Healthy Eating Index in medical students and against food frequency questionnaires in the general population [12]. Good test-retest reliability (*r =* 0.86) was reported in ethnically and educationally diverse groups [12]. In the current study, only nutrition questions were examined. Answers were coded according to previous studies with usually/often = 1, sometimes = 2, rarely/never = 3, and blank answers = 3 [13]. Questions are phrased so higher scores indicate healthier eating behaviors. The alcohol use answers were categorized by frequency of alcohol consumption over the past month. Frequency of consuming >1-2 drinks were categorized as 0–1 times = rarely/never(3), 1–6 times = sometimes(2), and >6 times = usually/often(1).

Body weight (to the nearest 0.5 lbs.) and height (to the nearest 0.5 inch) were collected during the athlete’s pre-participation physical examination. Waist circumference was obtained by using a standard tailor’s tape measuring the narrowest portion of the waist between the xyphoid process and naval, recorded to the nearest quarter inch and expressed in centimeters. Weight was measured on a laboratory scale.

### Data analysis

PCA was conducted with the first wave of data using the scree plot to determine the number of components to retain. EFA was conducted on the second wave of data to represent the realistic nature of the study measurement. Proportion of common variance >0.75 and chi-square significance test of retained factors against the inclusion of an additional factor were criteria used to determine the number of factors to retain. The second wave of athletes was surveyed to avoid dependency among the data. Last, a CFA, designed to test the fit of the exploratory factor model was performed. Factor score coefficients were obtained from the confirmed model output and scores were computed for each participant on each dietary pattern.

After progressing through the model identification steps to establish the construct validity of the REAP, male and female athletes were stratified by participation in aesthetic, or appearance-oriented sport; or non-aesthetic sport, in which success is not related to appearance. Aesthetic sports included gymnastics, swimming, diving, and wrestling. Non-aesthetic sports included golf, basketball, baseball, softball, soccer, football, volleyball, cross-country/track and field, water polo, and tennis. Mean differences between pattern scores were explored between aesthetic classification (aesthetic sport vs. non-aesthetic sport) for males and females using a two-way ANOVA. Regression prediction models to examine if an interaction between pattern scores and participation in aesthetic or non-aesthetic sport impact BMI and waist circumference were conducted. All data were analyzed using SAS 9.3 (Cary, NC) with significance set at *p <* 0.05.

## Results

Comparison of wave-1 (n = 150) and wave-2 (n = 241) (Table 1) showed that participants were similar across waves for age, gender, race, and aesthetic vs. non-aesthetic sport status.

**Table 1:** Descriptives of male, female, and total sample of 2 waves of data

***WAVE 1***				
		**Males (n=86)**	**Females (n-64)**	**Total (n=150)**
		**Mean SD**	**Mean SD**	**Mean SD**
Age		19.6 (1.4)	19.5 (1.2)	19.5 (1.3)
Height (cm)		183.4 (8.6)	169.9 (7.9)	177.6 (10.6)
Weight (kg)		87.3 (20.9)	67.4 (46.4)	78.8 (20.5)
BMI		25.8 (5.2)	23.2 (3.5)	24.7 (4.7)
		**N %**	**N %**	**N %**
Race				
	Caucasian	50 (33.3)	51 (34.0)	101 (67.3)
	African American	23 (15.3)	6 (4.0)	29 (19.3)
	Other	13 (8.7)	7 (4.7)	20 (13.3)
Sport				
	Aesthetic	28 (32.6)	13 (20.3)	41 (27.3)
	Non-aesthetic	58 (67.4)	51 (79.7)	109 (72.7)
***WAVE 2***				
		**Men (n=139)**	**Women (n=102)**	**Total (n=241)**
		**Mean SD**	**Mean SD**	**Mean SD**
	Age	20.0 (1.6)	19.1 (1.3)	19.6 (1.5)
	Height (cm)	186.3 (26.6)	170.1 (8.5)	179.4 (22.4)
	Weight (kg)	90.9 (20.8)	66.5 (10.3)	80.6 (21.0)
	Waist Circumference (cm)*	84.8 (9.1)	74.8 (7.5)	31.9 (3.9)
	BMI (kg/m2)	26.6 (5.1)	22.9 (2.5)	25.0 (4.5)
		**N %**	**N %**	**N %**
Race				
	Caucasian	82 66.13	73 80.22	155 72.09
	African-American	34 27.42	13 14.29	47 21.86
	Other	8 6.45	5 5.49	13 6.05
	Not Reported	15	11	26
Sport				
	Aesthetic	26 18.98	28 27.45	54 22.59
	Nonaesthetic	111 81.02	74 72.55	185 77.41
	Not Reported	2	0	2

### Principal components analysis (PCA)

A PCA oblique rotation (promax) was conducted on the 25 nutrition items of the wave-1 REAP. The initial analysis indicated seven components be retained based on eigenvalues >1 that explained 62.01% of the variance in the sample. The scree plot showed an inflection point suggesting five components be retained [14] that explained 53.2% of the data variance. Small communalities (<0.4) suggested that questions two (h^2^ = 0.31) and 28 (h^2^ = 0.34) be eliminated. Due to small loadings (<0.4) questions 22 (loading = 0.29) and 24 (loading = 0.22) were eliminated and cross loading (>0.35 on more than one factor) indicated questions 12 (loadings = 0.36, 0.35) and 13 (loadings = 0.38, 0.35) be eliminated. The final PCA resulted in 19 questions loading on five factors explaining 60.3% of the sample variance. Based on item factor loadings, factor one represented a dessert pattern (DES; sweets, dessert consumption), factor two represented a high-fat food pattern (FAT; fried foods, high-fat snack consumption), factor three represented a healthful eating pattern (HP; whole grain, fruit, vegetable consumption), factor four represented a meat choice pattern (MEAT; frequency, amount, and fat content of meats), and factor five represented a dairy pattern (DARY; whole milk, regular cheese, salad dressing consumption).

### Exploratory factor analysis (EFA)

Additional file 1: Table S2 displays the final rotated 5-factor pattern solution using 14 REAP items. The initial EFA on wave-2 data determined four factors should be retained based on proportion criterion (>0.75) although the chi-square was significant (χ^2^ = 165.2, *p <* 0.0001) indicating a rejection of the null-hypothesis (H_0_ = 4-factor model) and the testing of a 5-factor model. Low communalities on questions one (һ^2^ = 0.13), three (һ^2^ = 0.13), six (һ^2^ = 0.12), seven (һ^2^ = 0.24), 18 (һ^2^ = 0.32), and 23 (һ^2^ = 0.33) suggested they be eliminated from further analyses; but in keeping with the goal of achieving a simple solution (high loading on only factor with low loadings on all others), questions three (loading = 0.36) and seven (loading = 0.54) were retained. Questions 17, 18, and 23 were removed due to non-loading (<0.40). The EFA was rerun revealing model fit statistics (chi-square *p* > 0.05, Tucker-Lewis = 0.99) and the scree plot inflection point conducive to a 5-factor model with the 14 remaining variables. DES explained most of the shared variance and DARY, MEAT, HP, and FAT explained the remaining shared variance.

### Confirmatory factor analysis (CFA)

The wave-2 data was a good fit (RMSEA = 0.055, CFI = 0.934) to the 5-factor model with the 14 REAP items. The initial CFA conducted on the second wave of data showed the model to be good fit based on common fit indices (GFI = 0.936, CFI = 0.929, RMSEA = 0.058), however warning messages indicated fit statistics might not be accurate. A second-order CFA was conducted to examine the existence of a hierarchical model, but resulted in unclear factor score coefficients and worse model fit (GFI = 0.925, CFI = 0.906, RMSEA = 0.064). A multi-group CFA was conducted to determine if model fit improved with gender stratification. Fit indices indicated the gender-stratified model to be a slightly better fit overall (RMSEA = 0.055, CFI = 0.934), for males (GFI = 0.904), and females (GFI = 0.918). This gender-differentiated group structure was used based on improved fit indices (reported above). Pattern scores were computed by summing the product of each survey item score coefficient by the item’s numerical response.

### Pattern score differences, BMI and waist circumference

**Figure 1:**
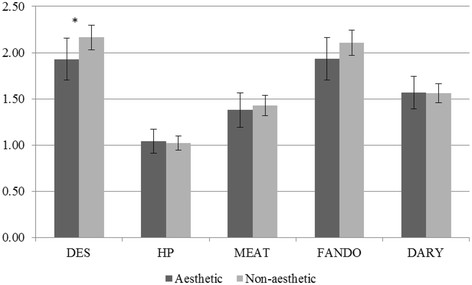
**Means and standard errors for dietary pattern scores of aesthetic and non-aesthetic sport male athletes.** All models adjust for age and race. *p < .05.

**Figure 2:**
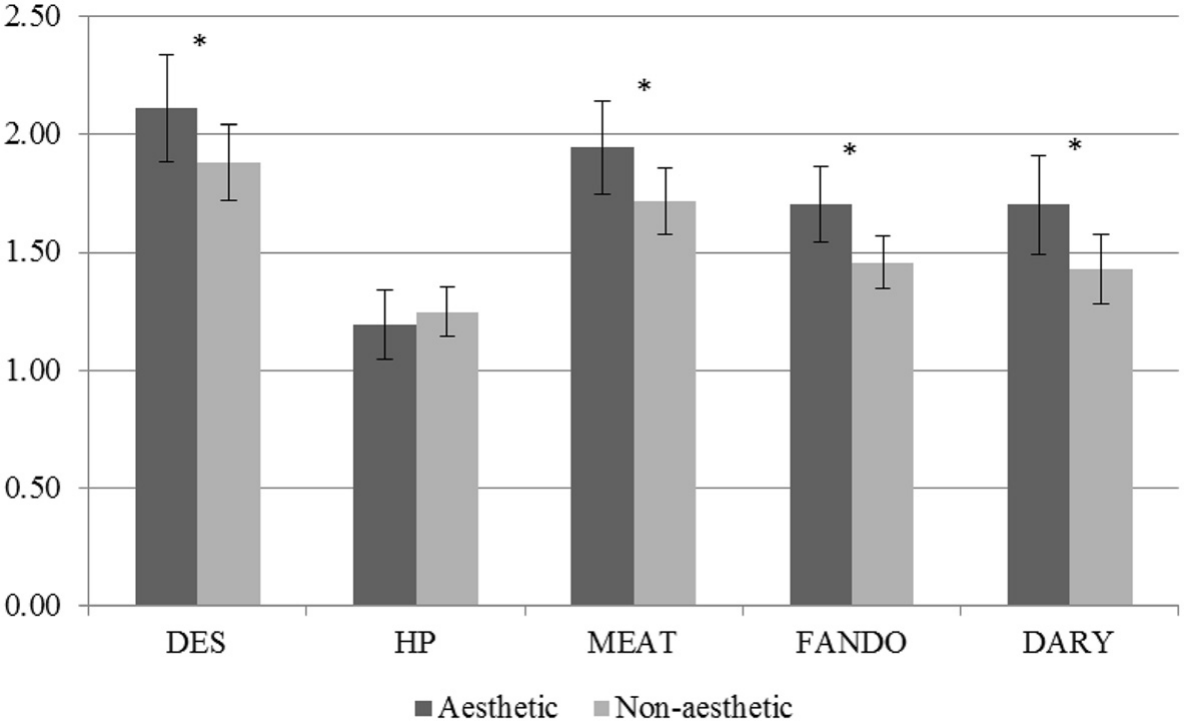
**Means and standard errors for dietary pattern scores of aesthetic and non-aesthetic sport female athletes.** All models adjust for age and race. *p < .05.

For males (Figure 1), a significant mean difference (*p <* .05) in DES pattern scores (mean ± SE) were observed between aesthetic (1.93 ± 0.11) and non-aesthetic sport (2.16 ± 0.07) athletes while controlling for age and race. No other significant differences were found in males. Figure 2 shows female aesthetic athletes had higher (better) scores compared to non-aesthetic female athletes for the DES (2.11 ± 0.11; 1.88 ± 0.08), MEAT (1.95 ± 0.10; 1.72 ± 0.07), FAT (1.70 ± 0.08, 1.46 ± 0.06), and DARY (1.70 ± 0.11, 1.43 ± 0.07) patterns while controlling for age and race. HP was not significantly different between female aesthetic and non-aesthetic athletes. Interactions between the pattern score and aesthetic/non-aesthetic sport in predicting BMI or waist circumference were not observed (*p* > .05).

## Discussion

Using pattern identification protocols, the REAP had construct validity for dietary pattern assessment in a population of NCAA athletes and distinguished different dietary habits between aesthetic and non-aesthetic athletes, particularly in females. Five factors were observed to reflect dietary intake: consumption of desserts, healthy foods, high-fat foods, dairy, and meat choices. Dietary patterns between aesthetic and non-aesthetic athletes were different in males and females. Aesthetic-sport males reported lower dessert pattern scores than non-aesthetic-sport males, while aesthetic-sport females reported higher pattern scores for the dessert, meat, high fat food, and dairy patterns. No interaction between dietary patterns and waist circumference and BMI were observed, indicating that the relationship between health metrics and pattern scores do not differ by sport type.

Several approaches can be used to measure individuals’ dietary patterns and multiple analyses should be used on multiple samples to verify the findings [15]. PCA is a useful screening procedure to reduce the initial pool of questions and trim those that do not contribute to eating patterns [15] while representing as much of the variation within the data as possible. EFA seeks to explore the number of factors underlying the data that best reproduce the correlations while accounting for error variance. PCA and factor analysis have been used previously to assess food intake patterns in relation to waist circumference and triglycerides [16], hence they are useful when examining associations between dietary patterns and health metrics. One approach to assessing diet is to examine intake compared to guidelines. However, our analysis took a data-driven approach, a method that has become acceptable over the past decade [10]. Using a series of multivariate analysis techniques, the underlying structure of this survey was determined in an under-studied yet high risk population of NCAA athletes [6].

The 5-factor solution is a unique finding among factor-analyzed dietary studies, possibly because college athletes’ eating behaviors are seldom examined using these methods. Most studies using the PCA/factor analysis approach involve middle-aged men and women and often find a limited amount of sample variance represented by components [8]. Our 5-factor PCA represented 60% of the sample variance. While variance accounted for is important in deriving dietary patterns, the interpretability of the solution is just as important [17]. Our solution is comparable to other studies in regards to pattern characteristics. Red meat consumption and vegetable/fruit intake patterns have been identified previously [18] as has a dairy pattern [19], but the dessert pattern has yet to be identified to our knowledge. Our results agree with previous studies concluding females have better diet scores than males [8], although this was evident in non-aesthetic sport females. Male non-aesthetic sport athletes had higher dessert, high-fat food, and dairy consumption scores than non-aesthetic sport females, indicating better eating choices for these three dietary patterns in this sub-group of male athletes.

In comparison to their recreational athlete and non-athletic counter parts, college athletes are at increased risk for poor dietary patterns. Lack of discipline, social obligations, time constraints, perception of the impact of a healthful diet, and ready access to healthful food are cited as barriers to healthful eating among college athletes [5]. Sports discipline is an important moderator when evaluating athlete nutrition, as unhealthful eating behaviors may be modeled from teammates [20]. Athletes often transition out of sport without adequate nutrition knowledge that may follow them for the rest of their lives [21], increasing risk of poor health outcomes.

There are some limitations to the data-driven approach to dietary pattern examination. Most studies use PCA, EFA, or CFA to derive latent factors. This study employed all three methods, a strength of the study. However, the patterns derived from these methods are not often predictive of a tangible outcome variable, such as BMI or waist circumference. This is likely due to the fact that while dietary patterns explain variation in eating behaviors, they are not specific to nor explain variation in nutrients consumed. The lack of variability in BMI (wave-1 SD = 4.7; wave-2 SD = 4.5) may have suppressed differences between dietary patterns as well. Specific to this population of college athletes, energy needs may not be the same across different types of sport. Therefore, a diet consisting of more higher-fat foods may be more appropriate in the more physically demanding sports. Other methods of analyses and specific diet composition measurement methods should be considered as a valuable alternative [22]. Also, bias may exist in the self-reporting of dietary habits, possibly contributing to under-reporting of unhealthful eating behaviors and over-reporting of healthier behaviors.

## Conclusions

The REAP demonstrated construct validity when measuring dietary patterns in a population of NCAA Division-I athletes. College athletes are a group that requires guidance in light of the increasing demands and expectations given dual roles as athlete and student. It is recommended that all athletes, regardless of sport, be screened for dietary intake behaviors. Education regarding healthful eating should be provided by a sport dietician to prevent unhealthful eating behaviors from being adopted. Young adults should continue to be monitored and advised on healthful dietary choices to encourage the development of healthful dietary habits that may persist into middle and late adulthood.

### Consent

Written informed consent was obtained from the patient for the publication of this report and any accompanying images.

## Competing interests

The authors declare no financial support for the work supported in the manuscript, sources of substantial technical assistance, or sources from which some or all of the data were taken.

## Authors’ contributions

JMK contributed to the acquisition of data, analysis and interpretation of data, drafting of the manuscript, and revising the manuscript for intellectual content. MPB contributed to the analysis and interpretation of the data and revising the manuscript for intellectual content. BEA contributed to the conception and design of the study and revising the manuscript for intellectual content. All authors read and approved the final manuscript.

## Additional file

## Supplementary Material

Additional file 1: Table S2. Exploratory factor analysis: rotated factor pattern of item loadings and communalities. Click here for file
