# Supplementary material for: Validity of the Rapid Eating Assessment for Patients for assessing dietary patterns in NCAA athletes
Source: J Int Soc Sports Nutr. 2014 Aug 15;11:42. doi: 10.1186/s12970-014-0042-y (PMC4190925; doi:10.1186/s12970-014-0042-y)
Supplement: Additional file 1: Table S2. — Exploratory factor analysis: rotated factor pattern of item loadings and communalities. [file s12970-014-0042-y-S1.doc]

Dessert (DES)

Healthful Foods

(HP)

Meat (MEAT)

High-fat Dairy

(DARY)

High-fat Food

(FAT)

Communalities

NUT02Q3

Eat less than 3 servings of whole grain products a day

0.02

0.37

-0.11

-0.02

-0.03

0.14

NUT02Q4

Eat less than 2-3 servings of fruit a day

-0.02

0.81

0.05

0.00

0.05

0.68

NUT02Q5

Eat less than 3-4 servings of vegetables/potatoes a day

0.00

0.76

-0.03

0.01

-0.04

0.56

NUT02Q7

Use 2% (reduced fat) or whole milk instead of skim (non-fat) or 1% (low-fat) milk?

-0.07

-0.04

-0.21

0.66

0.07

0.36

NUT02Q8

Use regular cheese (like American, cheddar, Swiss, Moterey Jack) instead of low fat or part

skim cheeses as a snack, on sandwiches, pizza, etc.

-0.05

0.02

0.20

0.65

-0.06

0.52

NUT02Q9

Eat beef, pork, or dark meat chicken more than 2 times a week?

0.03

0.02

0.70

0.03

-0.04

0.50

NUT02Q10

Eat more than 6 ounces of meat, chicken, turkey, or fish per day?

-0.15

-0.11

0.60

-0.12

0.03

0.33

NUT02Q11

Choose higher fat red meats like prime rib, T-bone steak, hamburger, ribs, etc..instead of lean

read meats

0.09

-0.02

0.54

0.04

0.10

0.38

NUT02Q14

Eat fried foods such as fried chicken, fried fish or French fries?

0.05

-0.02

0.05

0.01

0.91

0.91

NUT02Q15

Eat regular potato chips, nacho chips, corn chips, crackers, regular popcorn, nuts instead of

pretzels, low-fat chips or low-fat crackers, air-popped popcorn?

0.27

-0.01

0.01

0.22

0.34

0.41

NUT02Q16

Use regular salad dressing and mayonnaise instead of low-fat or fat-free sald dressing and

mayonnaise

0.13

0.07

0.16

0.37

0.02

0.30

NUT02Q19

Eat regular sweets like cake, cookies, pastries, donuts, muffins, and chocolate instead of low-

fat or fat-free sweets?

0.84

0.00

0.00

-0.11

-0.01

0.66

NUT02Q20

Eat regular ice cream instead of sherbet, sorbet, low-fat or fat-free ice cream, frozen yogurt,

etc.

0.66

-0.07

0.01

0.20

-0.09

0.49

NUT02Q21

Eat sweets like cake, cookies, pastries, donuts, muffins, chocolate and candies more than 2

times per day?

0.55

0.06

-0.07

-0.12

0.16

0.39

Variance Explained by each factor

2.24

1.62

1.73

1.76

1.96

% of Total Variance Explained by Each Factor

24.1

17.4

18.6

18.9

21.0

Shaded sections indicate item loadings on each factor

Additional file 1: Table S2. Exploratory Factor Analysis: Rotated Factor Pattern of Item Loadings and Communalities
